# Recombinant human TSH versus thyroid hormone withdrawal in adjuvant therapy with radioactive iodine of patients with papillary thyroid carcinoma and clinically apparent lymph node metastases not limited to the central compartment (cN1b)

**DOI:** 10.1590/2359-3997000000247

**Published:** 2017-01-27

**Authors:** Pedro Weslley Rosario, Gabriela Franco Mourão, Maria Regina Calsolari

**Affiliations:** 1 Santa Casa de Belo Horizonte MG Brasil Santa Casa de Belo Horizonte, MG, Brasil

**Keywords:** Papillary thyroid cancer, lymph node metastases, recombinant human thyroid, radioiodine

## Abstract

**Objective:**

To compare the short- and long-term outcomes of adjuvant therapy with radioactive iodine (RAI) preceded by the administration of recombinant human TSH (rhTSH) versus thyroid hormone withdrawal (THW) in patients with papillary thyroid carcinoma and clinically apparent lymph node metastases not limited to the central neck compartment (cN1b).

**Subjects and methods:**

The sample consisted of 178 cN1b patients at intermediate risk who underwent total thyroidectomy with apparently complete tumor resection [including postoperative ultrasonography (US) without anomalies] and who received adjuvant therapy with RAI (30-100 mCi) preceded by the administration of rhTSH (n = 91) or THW (n = 87).

**Results:**

One year after RAI, the rates of excellent response to therapy, i.e., nonstimulated thyroglobulin (Tg) ≤ 0.2 ng/mL with negative antithyroglobulin antibodies and negative neck US, and of structural disease were similar for the two preparations (84% and 4.5%, respectively, in both groups). During follow-up (median 66 months), the rate of structural or biochemical (nonstimulated Tg > 1 ng/mL, with increment) recurrence was also similar in the two groups (4.5%). In the last assessment, the percentage of patients without evidence of disease, i.e., nonstimulated Tg < 1 ng/mL and no evidence of structural disease, was similar for the two preparations [92.3% in the rhTSH group and 97.7% in the THW group (p = 0.17)].

**Conclusion:**

Preparation with rhTSH was equally effective (short- and long-term) as THW for adjuvant RAI therapy of cN1b patients at intermediate risk and with apparently complete tumor resection.

## INTRODUCTION

According to the American Thyroid Association (ATA), preparation with recombinant human TSH (rhTSH) stimulation is an acceptable alternative to thyroid hormone withdrawal (THW) for ablation of thyroid remnants with radioactive iodine (RAI) in patients with papillary thyroid carcinoma (PTC) at low or intermediate risk with lymph node (LN) metastases restricted to the central neck compartment (N1a) and discrete/microscopic metastases (1). This is considered a “strong recommendation” and “moderate-quality evidence” (1). Even more favorable, the European Thyroid Association (ETA) (2) and many authors (3,4) consider rhTSH to be the “preparation of choice” for these patients.

In contrast, in the absence of comorbidities that prevent THW, in intermediate-risk patients with LN metastases not limited to the central neck compartment (N1b) or clinically apparent/macroscopic metastases (cN1), rhTSH stimulation “may be considered” an alternative to adjuvant RAI treatment (1). Apparently, ATA does not consider rhTSH to be at the same level as THW (5). This observation is supported by the fact that this recommendation is considered a “weak recommendation” and “low-quality evidence” (1). In agreement, in the absence of any clinical contraindication, ETA (2) and other authors (3,4) also consider THW the most indicated preparation for patients with macroscopic LN metastases.

Indeed, ATA calls attention to the fact that, among the randomized studies comparing the efficacy of ablation with RAI preceded by rhTSH versus THW, few investigations included N1 patients (1). More importantly, almost all patients had exclusive central neck compartment metastasis (N1a) and the studies only reported the short-term outcomes (1). There was only one retrospective study that included a significant number of cN1b patients (6). This scenario has not changed since the publication of the ATA guidelines (1).

Obviously, more investigations, especially long-term studies, evaluating the efficacy of rhTSH specifically in these patients (cN1b) are necessary and desirable. The objective of this study was to compare the short- and long-term outcomes of adjuvant RAI therapy preceded by rhTSH versus THW specifically in patients with PTC and clinically apparent LN metastases not limited to the central neck compartment (cN1b).

## SUBJECTS AND METHODS

The study was approved by the Research Ethics Committee of our institution.

### Patients

Patients with PTC consecutively seen at our institution from 2006 to 2014 and undergoing total thyroidectomy with apparently complete tumor resection were first selected. Only patients with LN metastases detected by preoperative US or during intraoperative inspection by the surgeon [clinical N1 (cN1)] were submitted to LN dissection. A total of 206 patients had LN metastases outside the central neck compartment detected by preoperative US or during intraoperative inspection by the surgeon (cN1b). Eighteen patients with incomplete tumor resection, extensive extrathyroid invasion (pT4) and distant metastases known before RAI (detected by clinical examination or simple chest X-ray), who are classified as high risk by ATA (1), were excluded.

Therapy with levothyroxine (L-T4) was initiated immediately after surgery and the dose was adjusted to maintain TSH < 2 mIU/l. Neck US was performed 96 to 180 days (median 140 days) after thyroidectomy. Ten patients with positive neck US were excluded. Finally, 178 patients had US scans that showed no anomalies. These patients were treated with 30-100 mCi (1.1-3.7 GBq) RAI and composed the sample of this study.

### Adjuvant therapy with RAI

The patients were submitted to adjuvant RAI therapy after THW for 4 weeks or administration of rhTSH and of a low-iodine diet for 10 days prior to the procedure. Anterior and posterior whole-body images were obtained 7 days after therapy with RAI (RxWBS).

### Initial assessment after RAI therapy

The patients were evaluated 12 months after RAI therapy by the measurement of nonstimulated thyroglobulin (Tg), antithyroglobulin antibodies (TgAb), and neck US. In the case of patients with ectopic uptake on initial RxWBS, in addition to US, diagnostic WBS (DxWBS) and computed tomography (CT) were performed to exclude persistent disease. Imaging methods other than US [neck, chest and mediastinal CT, fluorodeoxyglucose-positron emission tomography (FDG-PET)/CT] were performed if nonstimulated Tg levels ≥ 1 ng/mL.

### Follow-up

The patients were maintained on TSH < 0.5 mIU/l and were followed up by clinical examination, measurement of nonstimulated Tg and TgAb at intervals of 6-12 months, and annual neck US. Imaging methods other than US were performed when nonstimulated Tg converted to levels ≥ 1 ng/mL. The time of follow-up ranged from 18 to 118 months (median 66 months).

### Outcomes

The outcomes were: (i) rate of excellent response to therapy, i.e., nonstimulated Tg ≤ 0.2 ng/mL, with negative TgAb and negative neck US, 1 year after RAI (1-4); (ii) structural disease 1 year after RAI; (iii) structural or biochemical (nonstimulated Tg > 1 ng/mL, with increment) recurrence during follow-up, and (iv) percentage of patients without disease in the last assessment, i.e., nonstimulated Tg < 1 ng/mL and no evidence of structural disease. In the case of patients with metastases detected by RxWBS who required a new cycle of RAI to treat persistent disease, the preparation (rhTSH or THW) was always the same in all cycles.

### Imaging methods

US was performed with a linear multifrequency transducer for morphological analysis (B-mode) and for power Doppler evaluation. All suspected lesions apparent on the scans (7,8) were evaluated by US-guided fine-needle aspiration biopsy. DxWBS was obtained 3 days after RAI administration (185 MBq) with rhTSH stimulation. Contrast-enhanced CT was performed on 5-mm sequential sections. FDG-PET/CT was carried out after stimulation with rhTSH. All images were analyzed by experienced Radiology or Nuclear Medicine specialists.

Apparent disease was defined based on the results of the imaging methods, cytology or histology, and/or unequivocal ectopic uptake (excluding false-positive results) on RxWBS or FDG-PET/CT.

### Assays

Chemiluminescent assays were used for the measurement of Tg [Access Thyroglobulin Assay, Beckman Coulter, Fullerton, CA (functional sensitivity of 0.1 ng/mL)] and TgAb [Immulite 2000, Diagnostic Products Corporation, Los Angeles, CA (reference value of up to 40 IU/mL) or ARCHITECT Anti-Tg, Abbott Laboratories, IL, USA (reference value of up to 4.11 IU/mL)]. Patients with positive TgAb were excluded.

### Statistical analysis

Means were compared between groups by the Student *t*-test or the nonparametric Mann-Whitney U test. Fisher’s exact test or chi-squared test was used to detect differences in the proportion of cases. A p value of less than 0.05 was considered to be significant.

## RESULTS

### Characteristics of the patients

The characteristics of the patients studied are shown in Table 1. The patients were classified as intermediate risk according to ATA (1). The two groups (rhTSH and THW) were similar in terms of sex, age, tumor stage, serum TSH immediately before the administration of ^131^I, RAI activity, frequency of metastases on initial RxWBS, and time of follow-up.

**Table 1:** Characteristics of the patients studied

	rhTSH (n = 91)	THW (n = 87)	p-value
Sex			
Male	23 (25.2%)	21 (24.1%)	1.0
Female	68 (74.5%)	66 (75.8%)	
Age [range (median), years]	18-76 (47)	18-72 (48)	0.93
Tumor stage (1)			
I	43 (47.2%)	41 (47.1%)	1.0
IVA	48 (52.7%)	46 (52.8%)	
Lymph node(s) with extranodal extension	37 (40.6%)	39 (44.8%)	0.65
Serum TSH [range (median), mIU/L]^a^	70-156 (105)	48-223 (98)	0.9
RAI activity			
30 mCi	39 (42.8%)	32 (36.8%)	0.44
50 mCi	14 (15.4%)	17 (19.5%)	
100 mCi	38 (41.7%)	38 (43.7%)	
Mean (mCi)	62.3	64.5	
Initial RxWBS			
Negative	86 (94.5%)	83 (95.4%)	1.0
Positive^b^	5 (5.5%)	4 (4.6%)	
Follow-up time [range (median), months]	24-118 (64)	18-118 (68)	0.9

rhTSH: recombinant human TSH; THW: thyroid hormone withdrawal; RAI: radioactive iodine; RxWBS: post-therapy whole-body scanning.

^a^ TSH immediately before the administration of ^131^I.

^b^ Uptake outside the thyroid bed and non-physiological: cervical (n = 3), mediastinal (n = 2), pulmonary (n = 2), cervical and pulmonary (n = 1), and cervical and mediastinal (n = 1).

### Initial assessment after adjuvant therapy with RAI

When evaluated 12 months after therapy with RAI, an excellent response to initial therapy was achieved in 149 patients (83.7%). Structural disease was detected in 8 patients (4.5%). Five of these patients had metastases on initial RxWBS and persistent disease (mediastinal in 2, pulmonary in 2, cervical and pulmonary in 1) and three had no metastases on initial RxWBS (in these cases, FDG-PET revealed bone metastases in 1, cervical in 1, and cervical and mediastinal in 1). The rates of excellent response and structural disease 1 year after initial therapy were similar in the rhTSH and THW groups (Table 2).

**Table 2:** Outcomes of patients submitted to adjuvant therapy with radioactive iodine preceded by rhTSH versus thyroid hormone withdrawal

	rhTSH (n = 91)	THW (n = 87)	p-value
Excellent response 1 year after initial therapy^a^	76 (83.5%)	73 (84%)	1.0
Structural disease 1 year after initial therapy	4 (4.4%)	4 (4.6%)	1.0
Recurrence during follow-up^b^	4 (4.4%)	4 (4.6%)	1.0
Structural recurrence	2 (2.2%)	4 (4.6%)	0.43
Biochemical recurrence^c^	2 (2.2%)	0	0.5
No evidence of disease in the last assessment^d^	84 (92.3%)	85 (97.7%)	0.17
Persistent disease in the last assessment^e^	7 (7.7%)	2 (2.3%)	

rhTSH: recombinant human TSH; THW: thyroid hormone withdrawal; RAI: radioactive iodine.

^a^ Nonstimulated Tg ≤ 0.2 ng/mL with negative antithyroglobulin antibodies and negative neck ultrasonography.

^b^ Patients without structural disease 1 year after RAI.

^c^ Nonstimulated Tg > 1 ng/mL, with increment, in the absence of apparent disease detected by the imaging methods.

^d^ Nonstimulated Tg < 1 ng/mL and no evidence of structural disease.

^e^ Nonstimulated Tg > 1 ng/mL or evidence of structural disease.

Twenty-one patients (11.8%) had elevated Tg in the absence of apparent disease detected by the imaging methods: 15 had indeterminate response [8/87 patients (9.2%) in the THW group and 7/91 patients (7.7%) in the rhTSH group (p = 0.8)] and 6 had incomplete biochemical response [2/87 patients (2.3%) in the THW group and 4/91 patients (4.4%) in the rhTSH group (p = 0.7)].

### Late follow-up

During follow-up, 8/170 patients (4.7%) without structural disease 1 year after RAI relapsed (Table 2). Six patients had structural disease (neck metastases detected by US in 3 and by FDG-PET in 1, and pulmonary metastases detected by CT and RxWBS in 1 and by CT and FDG-PET in 1). In 2 patients, nonstimulated Tg increased (> 1 ng/mL) in the absence of apparent disease detected by the imaging methods (US, CT, and FDG-PET). The recurrence rate was the same in the two groups (Table 2).

### Last assessment

All 162 patients without structural disease 1 year after RAI, who developed no recurrence and who were not submitted to any additional therapy, continued to have nonstimulated Tg < 1 ng/mL (≤ 0.2 ng/mL in 153), negative TgAb, and neck US showing no anomalies in the last assessment.

The two patients in whom Tg increased (biochemical recurrence) continued to have elevated Tg in the absence of structural disease identified until the last assessment.

In the last assessment, among the 14 patients with structural disease detected after initial therapy and who had undergone new surgery (LN dissection) and/or had been treated with RAI and/or external radiotherapy and maintained under TSH suppression, 7 patients achieved remission, structural disease persisted in 6 patients, and one patient had elevated Tg in the absence of structural disease. Thus, in the last assessment, the percentage of patients without evidence of disease did not differ between the two groups (Table 2). There was no case of death due to the tumor.

## DISCUSSION

Specifically in patients with PTC and clinical apparent lymph node metastases not limited to the central neck compartment (cN1b), when THW is not contraindicated, preparation with rhTSH for adjuvant RAI therapy is not yet considered to be at the same level as THW by ATA (1), ETA (2) and other authors (3,4). According to ATA, few randomized studies comparing the efficacy of ablation with rhTSH versus THW included N1 patients, almost all patients had exclusive central neck compartment metastasis (N1a), and only the short-term outcomes were reported (1). There was only one retrospective study that included a significant number of cN1b patients (6).

The present study included a significant number of patients (approximately 90 in each group) and not only evaluated the outcomes 1 year after RAI but also the long-term evolution of the patients. The median follow-up time was longer than 5 years. In this respect, it is known that 80% of recurrences occur in these first years (9-11). It should be noted that in the present study patients with elevated Tg without structural disease did not receive a new cycle of RAI. Consequently, the results of the last assessment of patients without persistent or recurrent disease reflect the effect of initial therapy. The study was prospective and the selection criteria were pre-defined. However, one limitation of our study is that the patients were not randomized, with the choice of the preparation being defined based on the access of the patient to rhTSH. Nevertheless, the groups were similar in terms of sex, age, tumor stage, TSH concentrations before RAI, RAI activity, and time of follow-up. In addition, the patients were followed up at the same institution and were submitted to the same follow-up protocol.

In the present study, rhTSH was equally effective as THW as a preparation for adjuvant therapy with RAI. The two preparations were also similar in the series of Hugo and cols. (6) including 183 N1b patients, but the efficacy of therapy was much lower than that found in the present series despite the use of higher RAI activities. In the study of Hugo and cols. (6), part of the cN1b patients exhibited additional findings [incomplete tumor resection, distant metastases, and extensive extrathyroid invasion (pT4)] and the efficacy of RAI was indeed higher when these patients were excluded and only intermediate-risk patients were considered. As supporting data, in a subsequent publication of the same group, the response rate of intermediate-risk cN1b patients to initial therapy was approximately 85% (12). In another series involving N1bM0 patients, the recurrence rate ranged from 15-20% (13-18), while this rate was approximately 10% in the present series. We attribute this apparent difference to the fact that the present study excluded cN1b patients who additionally exhibited incomplete tumor resection and extensive extrathyroid invasion (pT4) [high risk according to ATA (1)]. In addition, postoperative US (before RAI) was obtained for all patients of the present series as currently recommended (1) and patients with positive US were excluded. If this imaging method were not performed, these cases would have probably been detected subsequently, which would increase the rate of persistent or recurrent disease. Other smaller series that compared rhTSH versus THW for adjuvant RAI therapy included N1b patients, but the number of these patients was only about 10 in each group (19-22).

One limitation of this study was the lack of Tg results before RAI. However, specifically in these cN1b patients, negative Tg and TgAb do not exclude the need for RAI. First, ATA (1) and ETA (2) continue to recommend adjuvant therapy with RAI for cN1b patients, as do many authors (3,4). Second, in previous series, persistent disease was detected by RxWBS in 7% of N1 patients with stimulated Tg < 1 ng/mL and negative US (23) and in 12% of patients with undetectable nonstimulated Tg (24). It is possible that, if these patients had not received RAI, the lesions seen on RxWBS would have progressed to apparent disease during follow-up. Third, not every microscopic residual disease is apparent on RxWBS and yet can progress to structural disease. Destruction of these microscopic tumor foci, preventing recurrence, is exactly the rational of adjuvant therapy with RAI (1). In addition, the two groups (rhTSH and THW) were similar in terms of initial characteristics and patients were operated on by the same surgeons. The proportion of patients with negative pre-RAI Tg is therefore expected to be the same. Thus, the conclusion of the efficacy of adjuvant therapy with RAI in cN1b patients preceded by rhTSH or THW, which was the objective of the study, most likely would not be modified by this information.

Our results show that in patients with cN1b PTC without distant metastases and with complete tumor resection (including postoperative US without metastases), preparation with rhTSH was equally effective as THW for adjuvant therapy with RAI in the short- and long-term. Thus, rhTSH could be considered equal to THW also in these patients.

## References

[1] Haugen BR, Alexander EK, Bible KC, Doherty GM, Mandel SJ, Nikiforov YE, et al. 2015 American Thyroid Association Management Guidelines for Adult Patients with Thyroid Nodules and Differentiated Thyroid Cancer: The American Thyroid Association Guidelines Task Force on Thyroid Nodules and Differentiated Thyroid Cancer. Thyroid. 2016;26:1-133. 10.1089/thy.2015.0020 PMC4739132 26462967

[2] Lepoutre-Lussey C, Deandreis D, Leboulleux S, Schlumberger M. Postoperative radioactive iodine administration for differentiated thyroid cancer patients. Curr Opin Endocrinol Diabetes Obes. 2014; 21:363-71. 10.1097/MED.0000000000000100 25119656

[3] Schlumberger M, Pacini F, Tuttle RM. Initial treatment. In: Schlumberger M, Pacini F, Tuttle RM. 4th edition. Thyroid Tumors, 2015.

[4] Buffet C, Ghander C, le Marois E, Leenhardt L. Indications for radioiodine administration in follicular-derived thyroid cancer. Ann Endocrinol (Paris). 2015;76 (Suppl 1):S2-7. 10.1016/S0003-4266(16)30008-7 26826479

[5] Verburg FA, Aktolun C, Chiti A, Frangos S, Giovanella L, Hoffmann M, et al.; EANM and the EANM Thyroid Committee. Why the European Association of Nuclear Medicine has declined to endorse the 2015 American Thyroid Association management guidelines for adult patients with thyroid nodules and differentiated thyroid cancer. Eur J Nucl Med Mol Imaging. 2016;43:1001-5. 10.1007/s00259-016-3327-3 26883666

[6] Hugo J, Robenshtok E, Grewal R, Larson S, Tuttle RM. Recombinant human thyroid stimulating hormone-assisted radioactive iodine remnant ablation in thyroid cancer patients at intermediate to high risk of recurrence. Thyroid. 2012;22:1007-15. 10.1089/thy.2012.0183 22873801

[7] Rosario PW, de Faria S, Bicalho L, Alves MF, Borges MA, Purisch S, et al. Ultrasonographic differentiation between metastatic and benign lymph nodes in patients with papillary thyroid carcinoma. J Ultrasound Med. 2005;24:1385-9. 10.7863/jum.2005.24.10.1385 16179622

[8] Rosario PW, Tavares WC, Borges MA, Santos JB, Calsolari MR. Ultrasonographic differentiation of cervical lymph nodes in patients with papillary thyroid carcinoma after thyroidectomy and radioiodine ablation: a prospective study. Endocr Pract. 2014;20:293-8. 10.4158/EP13307.OR 24246348

[9] Kim WG, Yoon JH, Kim WB, Kim TY, Kim EY, Kim JM, et al. Change of serum antithyroglobulin antibody levels is useful for prediction of clinical recurrence in thyroglobulin-negative patients with differentiated thyroid carcinoma. J Clin Endocrinol Metab. 2008;93:4683-9. 10.1210/jc.2008-0962 18812478

[10] Brassard M, Borget I, Edet-Sanson A, Giraudet AL, Mundler O, Toubeau M, et al. Long-term follow-up of patients with papillary and follicular thyroid cancer: a prospective study on 715 patients. J Clin Endocrinol Metab. 2011;96:1352-9. 10.1210/jc.2010-2708 21389143

[11] Durante C, Montesano T, Torlontano M, Attard M, Monzani F, Tumino S, et al. Papillary thyroid cancer: time course of recurrences during postsurgery surveillance. J Clin Endocrinol Metab. 2013;98:636-42. 10.1210/jc.2012-3401 23293334

[12] Wang LY, Palmer FL, Nixon IJ, Tuttle RM, Shah JP, Patel SG, et al. Lateral neck lymph node characteristics prognostic of outcome in patients with clinically evident N1b papillary thyroid cancer. Ann Surg Oncol. 2015;22:3530-6. 10.1245/s10434-015-4398-2 PMC4977989 25665952

[13] O’Neill CJ, Coorough N, Lee JC, Clements J, Delbridge LW, Sippel R, et al. Disease outcomes and nodal recurrence in patients with papillary thyroid cancer and lateral neck nodal metastases. ANZ J Surg. 2014;84:240-4. 10.1111/ans.12045 23316684

[14] Kim SJ, Park SY, Lee YJ, Lee EK, Kim SK, Kim TH, et al. Risk factors for recurrence after therapeutic lateral neck dissection for primary papillary thyroid cancer. Ann Surg Oncol. 2014;21:1884-90. 10.1245/s10434-014-3507-y 24515566

[15] Hughes DT, Miller BS, Cohen MS, Doherty GM, Gauger PG. Outcomes of total thyroidectomy with therapeutic central and lateral neck dissection with a single dose of radioiodine in the treatment of regionally advanced papillary thyroid cancer and effects on serum thyroglobulin. Ann Surg Oncol. 2014;21:1647-52. 10.1245/s10434-013-3467-7 24385210

[16] Lee CW, Roh JL, Gong G, Cho KJ, Choi SH, Nam SY, et al. Risk factors for recurrence of papillary thyroid carcinoma with clinically node-positive lateral neck. Ann Surg Oncol. 2015;22:117-24. 10.1245/s10434-014-3900-6 25034816

[17] Jeon MJ, Kim WG, Jang EK, Choi YM, Song DE, Sung TY, et al. Sub-classification of lateral cervical lymph node metastasis in papillary thyroid carcinoma by pathologic criteria. PLoS One. 2015;10:e0133625. 10.1371/journal.pone.0133625 PMC4505852 26186205

[18] Park YM, Wang SG, Shin DH, Kim IJ, Son SM, Lee BJ. Lymph node status of lateral neck compartment in patients with N1b papillary thyroid carcinoma. Acta Otolaryngol. 2016;136:319-24. 10.3109/00016489.2015.1116045 26635131

[19] Melo M, Costa G, Ribeiro C, Carrilho F, Martins MJ, da Rocha AG, et al. Stimulated thyroglobulin at recombinant human TSH-aided ablation predicts disease-free status one year later. J Clin Endocrinol Metab. 2013;98:4364-72. 10.1210/jc.2013-2267 24037891

[20] Pitoia F, Abelleira E, Cross G. Recombinant human thyroid-stimulating hormone-aided remnant ablation achieves a response to treatment comparable to that with thyroid hormone withdrawal in patients with clinically relevant lymph node metastases. Eur Thyroid J. 2014;3:264-71. 10.1159/000369135 PMC4311299 25759804

[21] Wolfson RM, Rachinsky I, Morrison D, Driedger A, Spaic T, Van Uum SH. Recombinant human thyroid stimulating hormone versus thyroid hormone withdrawal for radioactive iodine treatment of differentiated thyroid cancer with nodal metastatic disease. J Oncol. 2016;2016:6496750. 10.1155/2016/6496750 PMC4763009 26977148

[22] Vallejo Casas JA, Mena Bares LM, Gálvez Moreno MA, Moreno Ortega E, Marlowe RJ, Maza Muret FR, et al. Thyroid remnant ablation success and disease outcome in stage III or IV differentiated thyroid carcinoma: recombinant human thyrotropin versus thyroid hormone withdrawal. Q J Nucl Med Mol Imaging. 2016;60:163-71. 26563902

[23] Nascimento C, Borget I, Al Ghuzlan A, Deandreis D, Chami L, Travagli JP, et al. Persistent disease and recurrence in differentiated thyroid cancer patients with undetectable postoperative stimulated thyroglobulin level. Endocr Relat Cancer. 2011;18:R29-40. 10.1677/ERC-10-0292 21183629

[24] Robenshtok E, Grewal RK, Fish S, Sabra M, Tuttle RM. A low postoperative nonstimulated serum thyroglobulin level does not exclude the presence of radioactive iodine avid metastatic foci in intermediate-risk differentiated thyroid cancer patients. Thyroid. 2013;23:436-42. 10.1089/thy.2012.0352 23067402

